# Bacteriomic Analyses of Asian Citrus Psyllid and Citrus Samples Infected With “*Candidatus* Liberibacter asiaticus” in Southern California and Huanglongbing Management Implications

**DOI:** 10.3389/fmicb.2021.683481

**Published:** 2021-07-02

**Authors:** Jiaquan Huang, Zehan Dai, Zheng Zheng, Priscila A. da Silvia, Luci Kumagai, Qijun Xiang, Jianchi Chen, Xiaoling Deng

**Affiliations:** ^1^Laboratory of Citrus Huanglongbing Research, College of Plant Protection, South China Agricultural University, Guangzhou, China; ^2^San Joaquin Valley Agricultural Sciences Center, Agricultural Research Service, United States Department of Agriculture, Parlier, CA, United States; ^3^Fundo de Defesa da Citricultura–Fundecitrus, Araraquara, Brazil; ^4^Plant Pest Diagnostic Center, California Department of Food and Agriculture, Sacramento, CA, United States; ^5^Jerry Dimitman Laboratory, Riverside, CA, United States

**Keywords:** “*Candidatus* Liberibacter asiaticus”, *Diaphorina citri*, metagenomic, bacteriomic, bacteriobiomic, HLB management, endosymbionts, endophytes

## Abstract

Citrus Huanglongbing (HLB; yellow shoot disease) is associated with an unculturable α-proteobacterium “*Candidatus* Liberibacter asiaticus” (CLas). HLB was found in southern California in 2012, and the current management strategy is based on suppression of the Asian citrus psyllid (*Diaphorina citri*) that transmits CLas and removal of confirmed CLas-positive trees. Little is known about Asian citrus psyllid-associated bacteria and citrus-associated bacteria in the HLB system. Such information is important in HLB management, particularly for accurate detection of CLas. Recent advancements in next-generation sequencing technology provide new opportunities to study HLB through genomic DNA sequence analyses (metagenomics). In this study, HLB-related bacteria in Asian citrus psyllid and citrus (represented by leaf midrib tissues) samples from southern California were analyzed. A metagenomic pipeline was developed to serve as a prototype for future bacteriomic research. This pipeline included steps of next-generation sequencing in Illumina platform, *de novo* assembly of Illumina reads, sequence classification using the Kaiju tool, acquisition of bacterial draft genome sequences, and taxonomic validation and diversity evaluation using average nucleotide identity. The identified bacteria in Asian citrus psyllids and citrus together included *Bradyrhizobium*, *Buchnera*, *Burkholderia*, “*Candidatus* Profftella armature,” “*Candidatus* Carsonella ruddii,” CLas, *Mesorhizobium*, *Paraburkholderia*, *Pseudomonas*, and *Wolbachia*. The whole genome of a CLas strain recently found in San Bernardino County was sequenced and classified into prophage typing group 1 (PTG-1), one of the five known CLas groups in California. Based on sequence similarity, *Bradyrhizobium* and *Mesorhizobium* were identified as possible source that could interfere with CLas detection using the 16S rRNA gene-based PCR commonly used for HLB diagnosis, particularly at low or zero CLas titer situation.

## Introduction

Citrus Huanglongbing (HLB; yellow shoot disease) is a highly destructive disease associated with a non-culturable α-proteobacterium, “*Candidatus* Liberibacter asiaticus” (CLas) (Jagoueix et al., 1994). Under field condition, CLas is transmitted by Asian citrus psyllid (ACP; *Diaphorina citri*) (Bové, 2006). In California, CLas was first detected in both ACP and citrus plants in 2012 in a residential area of the city of Hacienda Heights (Kumagai et al., 2013). Since then, CLas has been found in multiple residential areas, but not in citrus production fields despite active detection efforts, in southern California. As of the start of this project, the most recent CLas detection in both ACP and citrus was from San Bernardino county. To facilitate HLB research, selected genomes of CLas strains from California have been sequenced (Zheng et al., 2014a; Wu et al., 2015b; Dai et al., 2019). A prophage typing group (PTG) system that differentiates CLas strains in California has been established (Zheng et al., 2017; Dai et al., 2019).

Like other insects and plants, ACP and citrus also host microorganisms (microbiome, or bacteriome/bacteriobiome when bacteria are the focus). Examples of well-known ACP-associated bacteria (AABacts) are symbiotic “*Candidatus* Carsonella ruddii” and “*Candidatus* Profftella armature.” Both provide critical amino acids for ACP growth and development. Whole-genome sequences of these two symbionts have been published (Nakabachi et al., 2013; Wu et al., 2015a). Similarly, citrus-associated bacteria (CABacts) were also reported (Blaustein et al., 2017; Ginnan et al., 2018). CABacts identified after surface sterilization are referred to as endophytes, e.g., *Pantoea*, *Bacillus*, and *Curtobacterium* (Blacutt et al., 2020). The biological roles of most endophytic bacteria vary from beneficial to detrimental (Lodewyckx et al., 2002).

Recently, there has been increasing interests in research related on bacteriomes/microbiomes of plant diseases that could provide useful information for disease management. For example, grape Pierce’s disease (PD), caused by *Xylella fastidiosa* in California, was found to be effectively controlled by the inoculation of a bacterial endophyte, *Paraburkholderia phytofirmans* (Baccari et al., 2019). Strains of *Burkholderia* in citrus roots could trigger expression of disease-resistant genes (Zhang et al., 2017). CABacts were suspected to interfere with reliable detection of CLas with the commonly used TaqMan PCR protocol (HLBaspr-PCR, Li et al., 2006), particularly when samples contain a low titer of CLas (Ct value > 30) or no CLas (Shin et al., 2018; Bao et al., 2020).

Traditionally, plant bacteriome research utilized *in vitro* cultivation methodology. The use of next-generation sequencing (NGS) technology has extended the research to unculturable or not-yet-culturable bacteria. Studies on the microbiome associated with disease tissue begin with DNA extraction from pathogen-infected samples. The DNA is sequenced through an NGS platform such as Illumina to generate millions of short reads of 50–350 bp. Bacterial operational taxonomic units (OTUs) are created and tentatively named according to GenBank taxonomy. Typically, metagenomic programs such as Kaiju (Menzel et al., 2016), Kraken 2 (Wood et al., 2019), and MetaWRAP (Uritskiy et al., 2018) are used to assist the classification process.

It is also noted that mitochondria and chloroplasts are of prokaryotic origins and occur in high abundance (high copy number). Both mitochondria and chloroplasts have their own DNA genomes and could serve as sequence analysis controls in metagenomic experiments. Chloroplast DNA had a strong influence on CLas whole-genome assembly using Illumina MiSeq data (Zheng et al., 2014b). A metagenomic study of grape PD leaf samples revealed that chloroplast DNA was commonly found in putative bacterial reads (Van Horn et al., 2019). Filtering potential interfering sequences in an NGS data set before metagenomic analysis would improve the accuracy of sequence taxonomic classifications (Zhang et al., 2017).

This study explored the use of metagenomic technology to characterize the bacteriomes of HLB-associated ACP and citrus leaf samples collected from southern California. The objectives were as follows: (1) to establish a metagenomic pipeline to identify AABacts and CABacts and extract their draft genome sequences without chloroplast and/or mitochondrial sequence contamination, and the draft genome sequences were further cross-checked using GenBank bacterial whole-genome sequence database; (2) to evaluate the taxonomy status and diversity of AABacts and CABacts among the California samples collected from residential areas in southern California using average nucleotide identity (ANI) values, the current standard for bacterial species definition utilizing whole-genome sequences; and (3) to search for genome sequence evidence in AABacts and CABacts that could impact HLBaspr-PCR detection of CLas.

## Materials and Methods

### Asian Citrus Psyllid and Citrus DNA Samples

Asian citrus psyllid and citrus plant samples were collected from three geographically adjacent counties in southern California (Table 1). Collecting sites were in residential areas. Samples were collected by the California Department of Food and Agriculture or the California Citrus Research Board. DNA was extracted from individual ACP adults or citrus leaf midribs using the Qiagen MagAttract 96 DNA plant kit (QIAGEN Inc., Valencia, CA, United States) as described previously (Kumagai et al., 2013). The presence or absence of CLas in each sample was confirmed by HLBaspr-PCR targeting 16S rRNA gene (Li et al., 2006) and the PCR protocol targeting *nrdB* gene (Zheng et al., 2016), designated as RNRf-PCR.

**TABLE 1:** General information and selected metrics of next-generation sequencing data of Asian citrus psyllid (ACP) and citrus leaf DNA samples from southern California, United States.

No.	Sample	County	Host	Year of collection	Sequencing format	Read length (bp)	Total reads/total length (bp)	CLas Ct value^*a*^	References
1	A-SBCA19	San Bernardino	ACP	2019	NextSeq 500 1 × 75	35–85	507,362,298/43,125,795,330	23.39	This study
2	C-SBCA19	San Bernardino	Citrus	2019	NextSeq 500 1 × 75	35–85	468,054,578/39,784,639,130	26.78	This study
3	A-SBCA18	San Bernardino	ACP	2018	HiSeq 3000 2 × 100	101	482,412,866/48,723,699,466	28.62	This study
4	A-RSCA17	Riverside	ACP	2017	HiSeq 3000 2 × 100	101	471,308,572/47,602,165,772	29.80	This study
5	A-TECA18	Riverside	ACP	2018	HiSeq 3000 2 × 100	101	366,175,592/36,983,734,792	36.81	This study
6	A-AHCA17	Orange	ACP	2017	HiSeq 3000 2 × 150	150	611,118,478/91,667,771,700	23.31	Dai et al. (2019)
7	C-AHCA17	Orange	Citrus	2017	HiSeq 3000 2 × 100	101	593,133,332/59,906,466,532	27.52	Dai et al. (2019)

**Note* Ct was obtained according to HLBaspr-PCR (Li et al., 2006). ^*a*^CLas, “*Candidatus Liberibacter asiaticus.*”*

As shown in Table 1, samples 1, 2, and 3 were from San Bernardino County, the most recent location where CLas was confirmed at the time this research started. Sample 1 (A-SBCA19) was used as the ACP model for metagenomic pipeline development. Similarly, sample 2 (C-SBCA19) was used as a citrus model. Both samples 1 and 2 were from the same citrus tree. Sample 3, 4, and 5 had high HLBaspr-PCR Ct values (>28); and sample 5 (A-TECA18) was confirmed to be CLas negative by RNR-PCR; samples 6 and 7 were sequenced previously (Dai et al., 2019) but were used in this study as an ACP–citrus pair (Orange county) for comparison; another ACP–citrus pair was samples 1 and 2 from a different location (San Bernardino county).

### Development of Metagenomic Pipeline

#### Sample Preparation and Next-Generation Sequencing

Illumina sequencing (Illumina, Inc., San Diego, CA, United States) required > 1 μg of DNA. To meet this standard, 2–4 μl of sample DNA (mixture of bacteria + ACP or citrus hosts) was enlarged (i.e., increased all DNA simultaneously) with GenomiPhi^TM^ V2 DNA Amplification kit (GE Healthcare, Sigma-Aldrich Corp., St. Louis, MO, United States) following manufacturer’s instructions. The enlarged DNAs were sequenced by Illumina NextSeq or HiSeq formats (Table 1) through commercial sources. Only sequence reads with Q score > 30 were collected.

#### Acquiring Mitochondrial Genome (Mitogenome) and Chloroplast Genome Sequences

Illumina short reads were assembled into contigs (longer sequences) by MEGAHIT software version 1.1.2, a *de novo* assembler for metagenomics data (Li et al., 2016), with the default setting (–*k*-min = 21, –*k*-max = 99, –min-count = 2, –merge-level = 20, 0.98). MEGAHIT contigs of ACP mitogenome and citrus mitogenome and chloroplast genome were first identified by BLASTn (Camacho et al., 2009) search (identity > 95% and *e*-value > 1E-64) referenced to DQ864733.1 (citrus chloroplast genome, Bausher et al., 2006), NC_037463.1 (citrus mitogenome, Yu et al., 2018), and KY426014.1 (ACP mitogenome, Wu et al., 2017). Sample mitogenome and chloroplast genome sequences were then collected using the criteria of >99% identity and >200 bp in alignment length.

#### Filtering Sequences of Host Mitochondria, Chloroplasts, and Chromosomes

Illumina short reads of each ACP or citrus samples were filtered using Bowtie 2 software (Langmead and Salzberg, 2012) to remove sequences of host mitochondria, chloroplasts, and chromosomes. For mitochondrial and chloroplast DNA filtering, the corresponding sequences acquired as mentioned above were used as references. For host chromosomal DNA filtering, the ACP whole genome (Diaci V3 with the removal of No. 9 chromosomal scaffold containing bacterial symbiont sequences, available in https://citrusgreening.org/, Hosmani et al., 2019), *Citrus sinensis* genome (AJPS00000000.1, Xu et al., 2013), and *Citrus clementine* genome (AMZM00000000.1, Wu et al., 2014) were used as references. The filtered reads of each ACP and citrus samples were assembled in contigs by MEGAHIT software version 1.1.2, with the default setting mentioned above.

#### Acquisition of Asian Citrus Psyllid-Associated Bacteria and Citrus-Associated Bacteria Draft Genome Sequences

The filtered MEGAHIT contigs of each sample were classified by the Kaiju program using greedy mode (-a greedy -e 2 -m 20 -E 1E-5) against the bacterial RefSeq database (version 94). The classified contigs were collected according to the proposed OTUs at the genus level. The sequences were considered as the draft genome sequence version 1. The final version was generated after validation and removal of redundant sequences using CD-HITest at identity cutoff of 95% and coverage threshold of 0.0 (Huang et al., 2010).

#### Draft Genome Sequence Evaluation

The bacterial nature of each contig in a draft genome version 1 was evaluated through standalone BLASTn search against the genome sequences of the corresponding bacterial genus in GenBank RefSeq database (version 99) with the following parameters: word size = 28, *e*-value = 1E-64, and cover length > 200 bp, with identity > 80%. For the non-BLASTn matched contigs, BLASTx search was further used since protein sequences were evolutionally more conserved. The BLASTx parameters were as follows: word size = 3, *e*-value = 1E-64, cover length > 30 amino acid, and similarity > 40%. All BLASTn and BLASTx confirmed contigs were collected as the final version of the draft genome sequences. Assessment metrics of draft genome sequences were obtained using QUAST version 16 (Gurevich et al., 2013).

#### Bacterial Taxonomy Evaluation

The Kaiju taxonomy assignments of each OTU were evaluated by calculating ANI (Konstantinidis and Tiedje, 2005) using program pyani version 0.1.3.2 (Pritchard et al., 2016) with fragment size of 500 bp. From each OTU (at genus level), the corresponding whole-genome sequences in GenBank RefSeq database were used for pairwise ANI calculations. Bacterial genome ANIs were also calculated among the seven ACP/citrus samples for inter-strain/inter-sample diversity evaluations. To estimate average nucleotide coverage (ANC), Illumina reads from each sample were mapped to the draft genome using BBMAP program^1^.

### Characterization of “*Candidatus* Liberibacter asiaticus” Strains

The draft genome sequences of all CLas strains were characterized following the outline of Dai et al. (2019), which included prophage typing, and descriptions of loci *terL* (phage DNA terminase large subunit, Deng et al., 2014), *trn* (tandem repeat number at open reading frame CLIBASIA_01645, Chen et al., 2010), and miniature inverted-repeat transposable elements (MITEs; Wang et al., 2013). To detect the circularity of prophage sequence, the method of Zheng et al. (2018) involving identification of related *de novo* contigs and read walking (Shih et al., 2019) was used.

CLas strain A-SBCA19 was further used to acquire a more complete whole-genome sequence on top of the draft genome sequence from the Kaiju collection of MEGAHIT contigs. The process primarily combined the assemblies of reference-mapping and *de novo* assembly of both NextSeq data (Table 1) and the data from an additional Illumina sequencing (HiSeq 2000, a total of 20 Gbp) as reported previously (Wu et al., 2015b; Zheng et al., 2017). The improved A-SBCA19 genome sequence was annotated using RAST webserver (Aziz et al., 2008).

### *In Silico* Investigation of Asian Citrus Psyllid-Associated Bacteria and Citrus-Associated Bacteria Sequences Related to HLBaspr-PCR

Because *Bradyrhizobium* and *Mesorhizobium* were detected in all ACP samples by Kaiju analysis, which by design focused on protein coding sequences, rRNA gene sequences might not be included in the draft genome. As a correction effort, the 16S rRNA gene sequences of *Mesorhizobium terrae* str. NIBRBAC000500504 (NZ_CP044218.1) and *Bradyrhizobium* SK17 (NZ_CP025113.1) that had the highest ANI values in A-SBCA19 sample were downloaded from GenBank database and used as references for bowtie 2 mapping with sensitive parameter (-D 15 -R 2 -N 0 -L 22 -i S,1,1.15) using Illumina short reads of each sample. Reads similar to HLBas/HLBp/HLBr region were identified, assembled, and aligned with manual justifications.

### TaqMan PCR Procedures

TaqMan PCR was performed on an Applied Biosystems Step One Plus Real-Time PCR System. PCR was performed in 20-μl volume reactions consisting of the following reagent: 10 μl of Fast Universal PCR Master Mix (2×) (Applied Biosystems, Foster City, CA, United States), 1 μl of DNA template (25 ng), 0.2 μl of TaqMan probe (5 μM), and 0.4 μl of each forward and reverse primer (10 μM). The primer/probe sets were HLBas/HLBp/HLBr (Li et al., 2006) and RNR1f/RNRp/RNR1r (Zheng et al., 2016). The standard amplification procedure started at 95°C for 20 s, following by 40 cycles at 95°C for 10 s and 60°C for 20 s. The fluorescence signal was captured at the end of each 60°C step. The data were analyzed in Step One plus software (version 2.3, Applied Biosystems).

## Results

A total of seven sets of Illumina sequencing data ranging from 36 to 91 Gbp were collected (Table 1). Each data set was considered as a pro-metagenome for further analyses to generate a set of bacterial draft genome sequences (bacteriome). With the NextSeq data from sample A-SBCA19 (representing ACP) and the NextSeq data from C-SBCA19 (representing citrus), a pipeline from DNA preparation to acquisition of draft bacterial genome sequences was presented in Table 2. The pipeline was used to acquire bacterial draft genome sequences from the other five data sets/pro-metagenomes, i.e., A-SBCA18, A-RSCA17, A-TECA18, A-AHCA17, and C-AHCA17.

**TABLE 2:** A pipeline to acquire draft genome sequences of ACP-associated bacteria (AABacts; represented by sample A-SBCA19) and citrus-associated bacteria (CABacts; represented by sample C-SBCA19).

Step	Action	Results
1	Extraction of ACP/citrus midrib DNA and MDA amplification	DNA preparation
2	High throughput sequencing (Illumina HiSeq, NextSeq)	Short reads
3	Filtering short reads using Bowtie 2 with (1) ACP mitogenome and whole-genome sequences (2) Citrus chloroplast genome, mitogenome, whole-genome sequences	Presumably AABacts or CABacts enriched short reads
4	*De novo* assembly using MEGAHIT software	Presumably AABacts or CABacts long contigs (MEGAHIT contigs)
5	Kaiju taxonomic classification of MEGAHIT contigs referenced to GenBank Refseq database	Operational taxonomic units (OTUs) with GenBank taxonomy to genus level
6	Extraction of Kaiju contigs from each OTU	Version 1 of draft bacterial genome sequences
7	Validation of sequences in draft genome sequence version 1 using BLASTn and BLASTx against corresponding bacterial whole-genome sequences in GenBank RefSeq database	Final version of draft bacterial genome sequences
8.	Calculation of average nucleotide identity (ANI) between the final version of draft genome sequences and the whole-genome sequences of the corresponding bacterial genus in GenBank RefSeq database	Taxonomic conclusion of AABacts and CABacts

**Note* Computer scripts are available at request.*

**FIGURE 1:**
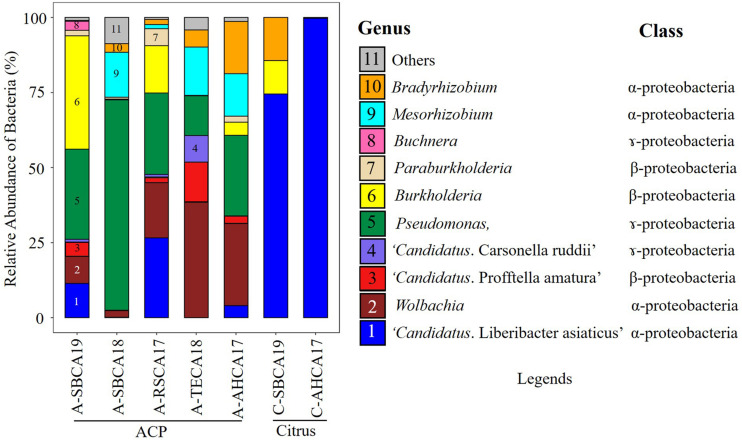
Relative abundance of bacteria (%) in Asian citrus psyllid (ACP)/citrus samples from southern California. The relative abundance was calculated based on the Kaiju classification of top 10 ranked genera from *de novo* assembled (MEGAHIT) contigs filtered by mitogenome, chloroplast genome (citrus only), and host genome. Bacterial names were coded with colors and numbers along with their class and shown in legends.

**FIGURE 2:**
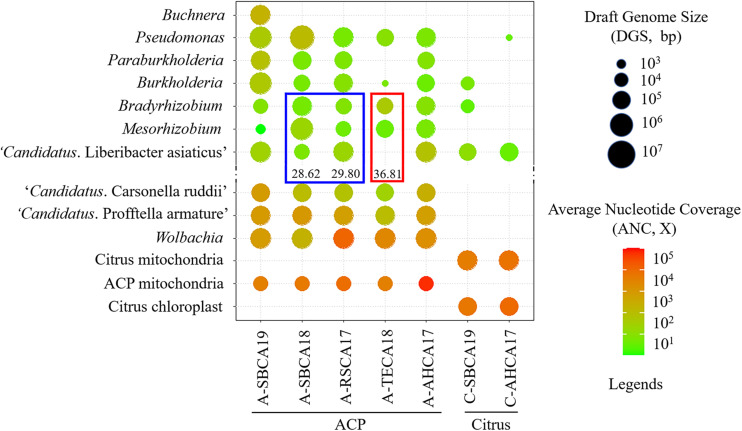
Graphical representation of draft genomes of ACP-associated bacteria (AABacts) and citrus-associated bacteria (CABacts) as listed in Supplementary Tables 1, 2. Draft genome size (DGS) is coded by circle sizes; average nucleotide coverage (ANC) is coded by colors. The blue box highlights the comparison of three bacteria in two samples with “*Candidatus* Liberibacter asiaticus” (CLas) in TaqMan HLBaspr-PCR Ct values near 30. The red box highlights the comparison of three bacteria in a single sample where CLas was not detected but shows TaqMan HLBaspr-PCR Ct values.

Ten bacterial genera were selected based on the contigs abundance from Kaiju classification results to describe the seven bacteriomes (i.e., seven samples) (Figure 1). These were *Bradyrhizobium*, *Buchnera*, *Burkholderia*, “*Ca*. Carsonella ruddii,” CLas, “*Ca*. Profftella armature,” *Mesorhizobium*, *Paraburkholderia*, *Pseudomonas*, and *Wolbachia*. The five ACP bacteriomes had all 10 bacteria. The two citrus bacteriomes had only four bacteria: CLas, *Burkholderia*, *Bradyrhizobium*, and *Pseudomonas* (Figure 2).

The final versions of draft genome sequences of all AABacts and CABacts are shown in Supplementary Table 1. The mitogenome and chloroplast genome sequences are shown in Supplementary Table 2. Figure 2 graphically summarizes the data in Supplementary Tables 1, 2. Each draft genome was described by five metrics: draft genome size (DGS), RC% (percentage of DGS/Reference genome size), Total contig in number, N50 in bp, and ANC. RC% provided an estimate of a genome completeness related to the arbitrarily selected reference genome. Noted that no CLas sequences was detected in A-TECA18 (Ct = 36.81). *Buchnera* was found only in A-SBCA19.

**FIGURE 3:**
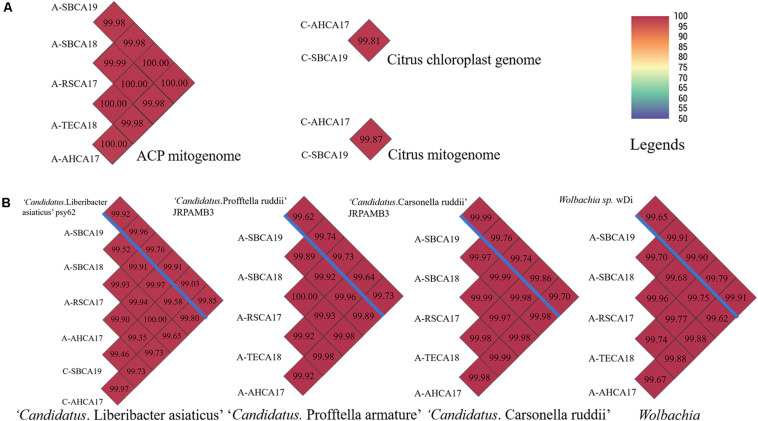
Pairwise average nucleotide identity (ANI) comparisons of **(A)** mitogenomes of ACP samples and mitogenome and chloroplast genome of citrus samples and **(B)** draft genomes of “*Candidatus* Liberibacter asiaticus,” “*Candidatus* Profftella armature,” “*Candidatus* Carsonella ruddii,” and *Wolbachia*, from ACP and citrus samples from southern California. Draft genome sizes were listed in Supplementary Tables 1, 2. **(B)** Reference genomes were listed above the blue bar. ANI values were also coded in color as explained in the legends. ANI was calculated based on fragment size of 500 bp.

Pairwise ANI comparisons of mitogenomes and chloroplast genomes are shown in Figure 3A. ANIs of all ACP mitogenomes were >99.00. Similarly, the two citrus samples showed a high degree of relatedness in their mitogenomes and chloroplast genomes (ANI > 99.00). ANIs of CLas and ACP endosymbionts are shown in Figure 3B. The ANIs were all >99.00, confirming the species status of these bacteria from different samples. The genome sequences of ACP endosymbionts have been deposited in the National Center for Biotechnology Information (NCBI) with GenBank accession numbers listed in Supplementary Table 1.

**FIGURE 4:**
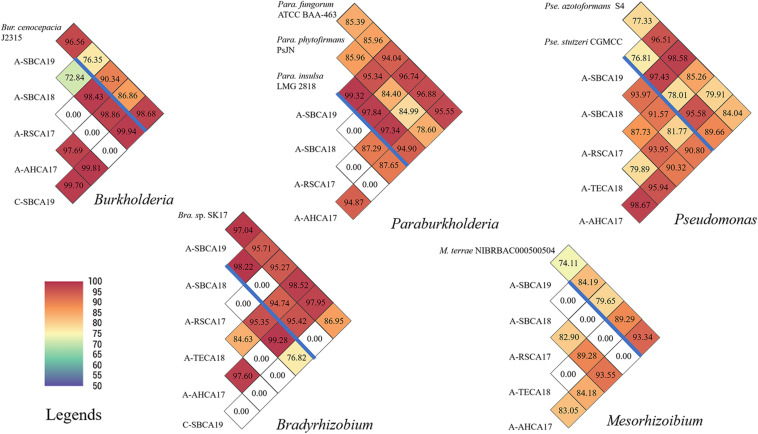
Pairwise ANI comparisons of draft genomes of *Burkholderia*, *Paraburkholderia*, *Pseudomonas*, *Bradyrhizobium*, and *Mesorhizobium* strains from ACP and citrus leaf samples from southern California. Draft genome sizes are listed in Supplementary Table 2. Reference genomes are listed above the blue bar. ANI values are also coded in color as explained in the legends. ANI was calculated based on fragment size of 500 bp.

In contrast, pairwise ANI values of the five AABacts and CABacts revealed significant inter-strain/inter-sample variations (Figure 4). For all the five bacteria genera from different samples, many ANIs were below the species threshold of 95, suggesting the presence of different species, or even different genera. An ANI of 0.00 represented that the draft genomes are too small to compare or ANI < 70, the program threshold. For the convenience of discussion, these bacteria were named only by the genus names, i.e., *Bradyrhizobium*, *Burkholderia*, *Mesorhizobium*, *Paraburkholderia*, and *Pseudomonas*. The draft genome sequences of these bacteria and the single sample *Buchnera* have been deposited in NCBI and identified by contig numbers under a common GenBank accession number for each bacteriome (Supplementary Table 1).

Table 3 summarizes the analyses on prophage typing and variations of CLas strains at the selected loci (*terL*, MITE, and *trn*). The final draft genome of CLas strain A-SBCA19 was 1,186,882 bp (81 contigs) with a Type 1 prophage that had no detected circular plasmid form. This recent CLas strain from San Bernardino County belonged to PTG-1, one of the five reported CLas groups in California (Dai et al., 2019). The genome sequence of CLas strain A-SBCA19 has been deposited in the NCBI under the GenBank accession number JADBIB000000000.1.

**TABLE 3:** Genomic characterization and comparisons of “*Candidatus* Liberibacter asiaticus” (CLas) strains from ACP or citrus samples collected from three counties in southern California.

CLas strain	CLas draft genome size (bp)/contigs/N50	PTG (prophage type group)/prophage sequence (bp)/circularity	*terL* group	MITE type	*trn* type (repeat number)	References
San Bernardino county						
A-SBCA19	1,186,882/81/27,492	1/33,553/no	Asiatic	B1	<10 (6)	This study
C-SBCA19	349,326/959/338	1/38, 221/yes	Asiatic	A, B2	Not found	This study
A-SBCA18	173,412/1,229/106	1/6,886/no	Asiatic	Not found	Not found	This study
Riverside county						
A-RSCA17	964,918/705/5,117	1/37,300/no	Asiatic	A, B2	<10 (8)	This study
A-TECA18	CLas not detected	Not applied	Not applied	Not applied	Not applied	This study
Orange county						
A-AHCA17	1,229,739/1/1,229,739	1/38,730/yes	Asiatic	B1	<10 (7)	Dai et al. (2019)
C-AHCA17	925,768/3,696/286	1/22,589/no	Asiatic	B1	<10 (8)	Dai et al. (2019)

As shown in Table 3, Type 1 prophages were also detected in the other CLas strains. However, circular plasmid forms of the prophages were only detected in strain C-SBCA19 (38,221 bp) and A-AHCA17 (38,730 bp). In strain C-SBCA19, the CLas chromosomal sequence was partial (98,097 bp, RC% = 7.99), whereas in strain A-AHCA17, CLas chromosomal sequence was complete (1,222,637 bp, RC% = 99.10).

Sequences homologous to HLBas/HLBp/HLBr (primers/probe sequences) were detected in *Mesorhizobium* and *Bradyrhizobium*, in both selected reference genomes and draft genomes of AABacts (Table 4). Sequence comparisons showed that there existed nucleotide mismatches in HLBas and HLBr regions but none in HLBp region. Although not experimentally tested, it was believed that these *Mesorhizobium* and *Bradyrhizobium* sequences were similar enough to generate some level of DNA amplification in the HLBaspr-PCR, particularly in the absence of CLas and when the titers of *Mesorhizobium* and *Bradyrhizobium* were high.

**TABLE 4:** Nucleotide sequence comparisons of HLBas/HLBp/HLBr locus among CLas strain A4, *Bradyrhizobium* and *Mesorhizobium* strains shown in Figure 2, and representative strains of *Mesorhizobium terrae* str. NIBRBAC000500504 and *Bradyrhizobium* sp. SK17.

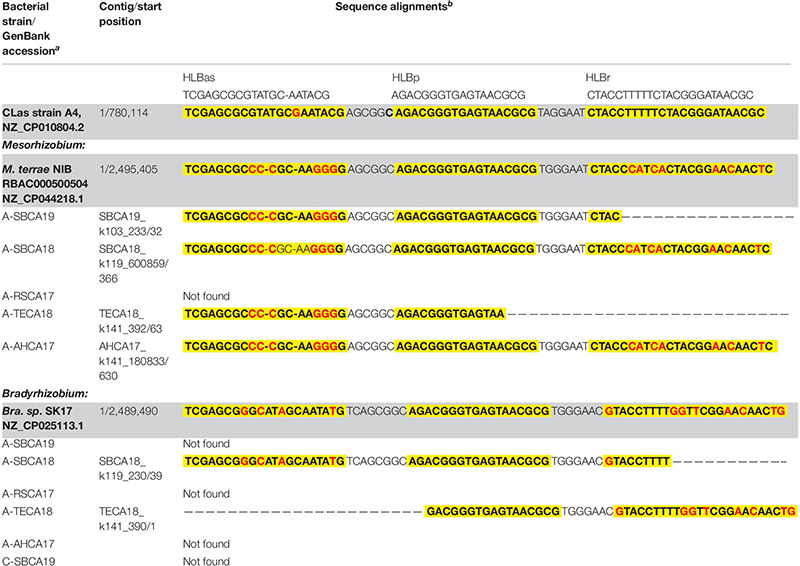

*^*a*^Gray shading highlights the reference strains.*

*^*b*^Yellow highlights are regions aligned with HLBas, HLBp, and HLBr. Red letters indicate mismatched nucleotides.*

## Discussion

We developed a metagenomic pipeline (Table 2) and used it to identify and characterize 10 AABacts/CABacts at the draft whole-genome sequence level (Figure 2). This research is unique and an extension of the HLB metagenomic research based on a single *rrn* locus (Zhang et al., 2013; Kolora et al., 2015; Blaustein et al., 2017; Ginnan et al., 2018; Meng et al., 2019; Song et al., 2019). As anticipated, the metagenomic pipeline successfully assembled the sequences of ACP and citrus mitogenome, and citrus chloroplast genome (Supplementary Table 2). Similarly, genome sequences of ACP endosymbionts were effectively acquired with RC% mostly over 90 and the lowest at 55 (Supplementary Table 2). All these indicate the reliable performance of the metagenomic pipeline (Table 2).

Draft genome sequence analyses provided new information that facilitates our understanding of HLB biology and management. However, research challenges remain, particularly for the AABacts and CABacts that could only be named at the genus level (Figure 2), along with their significant sequence variations to reference genomes and among different samples (Figure 4).

### Burkholderia

To our knowledge, *Burkholderia* has not been identified in ACP. The biological role of this bacterium is not clear. The bacterium has been reported in citrus root samples (Trivedi et al., 2010; Zhang et al., 2017), and it may not be surprising to find them in leaves. The ACP strain (A-SBCA19) and the citrus strain (C-SBCA19), both from San Bernardino county, were highly similar (ANI = 99.94), suggesting that they were the same species or even the same strain. This implies that ACP might have acquired *Burkholderia* from the infected citrus tree in San Bernardino county.

### Paraburkholderia

Similar to *Burkholderia*, *Paraburkholderia* has not been identified in ACP. Unlike the case of *Burkholderia*, *Paraburkholderia* was not detected in citrus sample C-SBCA19 or in C-AHCA17 (Figure 2). However, we could not exclude the possibility that the bacterial titer was too low to be detected by the technique used. The same explanation could be applied to the absence of *Paraburkholderia* in the ACP sample A-TECA18 (Figure 2). It is interesting that *Paraburkholderia* strain A-SBCA19 was highly similar to *Pa. phytofirmans* strain PsJN (ANI = 95.34, Figure 4). Strain PsJN showed its biocontrol capacity grape PD (Baccari et al., 2019).

### Pseudomonas

All ACP samples had *Pseudomonas* bacteria in relatively high abundance (Figure 2). A 16S rRNA gene-based study identified *Pseudomonas* from an egg through all nymph stages of ACP in China (Meng et al., 2019). In this study, *Pseudomonas* was also detected in at least one citrus sample (C-AHCA17, Figure 2). An interesting observation was that, in ACP, an *in vitro* culture method detected *Pseudomonas putida*, yet the 16S rRNA gene-based 454 pyrosequencing approach did not reveal the presence of *Pseudomonas* (Kolora et al., 2015). It is noted that ACP sample A-SBCA18 had a very large DGS (>30 Mbp, Figure 2 and Supplementary Table 1). This suggests that the sample could contain a mixture of significantly different *Pseudomonas* species. As shown in Figure 4, DGS of the A-SBCA18 *Pseudomonas* is highly similar to that of *Pseudomonas azotoformans* S4 (ANI = 98.58) and that of *Pseudomonas stutzeri* CGMCC (ANI = 97.43). However, *Pse. azotoformans* S4 and *Pse. stutzeri* CGMCC are significantly different (ANI = 77.33). The exact taxonomy details of these pseudomonads remain to be studied in the future.

The HLBaspr-PCR (Li et al., 2006) has widely been used in HLB diagnosis. It has long been suspected that some AABacts and CABacts could contribute to the high Ct values of the PCR results (Bao et al., 2020). Shin et al. (2018) isolated *Bradyrhizobium* from citrus roots and demonstrated that the bacterium interfered with HLBaspr-PCR. The detection of *Bradyrhizobium* and the closely related *Mesorhizobium* prompted us to investigate the nucleotide sequence evidence of HLBaspr-PCR interference.

### Bradyrhizobium/Mesorhizobium

The roles of *Bradyrhizobium* and *Mesorhizobium* bacteria in ACP are not known. Current knowledge about these bacteria is mostly limited to their nitrogen-fixing capacity in plant roots (Sawada et al., 2003). Differences between CLas and *Bradyrhizobium*/*Mesorhizobium* at the HLBas/HLBp/HLBr locus are mainly nucleotide mismatches and deletions, eight in HLBas and seven in HLBr (Table 4). This level of mismatches would make *Bradyrhizobium*/*Mesorhizobium* DNA not as competitive as CLas DNA to serve as template for HLBaspr-PCR. Therefore, at high CLas titer sample, the influence on *Bradyrhizobium*/*Mesorhizobium* is minimal. However, in the absence of CLas (sample A-TECA18, red box in Figure 2), high Ct values (36.81) could be generated, which might be interpreted as false positives.

Another situation in which *Bradyrhizobium* and *Mesorhizobium* bacteria could impact HLBaspr-PCR is the reduction of Ct values when CLas titers are low. As shown in the blue box in Figure 2 and Supplementary Table 1, A-SBCA18 had a lower Ct value (28.62) but smaller CLas DGS (19,674 bp), whereas, A-RSCA17 had a higher Ct value (29.80) but larger CLas DGS (799,019 bp). This could be explained by the higher level of *Mesorhizobium* DGS (5,990,225 bp) and *Bradyrhizobium* DGS (464,062 bp) in the A-SBCA18 sample than that in the A-RSCA17 sample (21,714 and 26,967 bp, respectively). Therefore, at low CLas titers (Ct values around 30 in this study), high titers of *Mesorhizobium* and *Bradyrhizobium* could inflate the true CLas titer using HLBaspr-PCR. Note that partial CLas genome assembly is associated with low CLas titer in a sample based on our past CLas genome sequencing experience.

### Buchnera

*Buchnera* has never been reported in ACP. It was a surprise that the bacterium was detected and only detected in sample A-SBCA19. Among the 63 *Buchnera* whole-genome sequences in GenBank Refseq database (version 99), the highest ANI was 86.49 with *Buchnera aphidicola* str. Afa-UT1. The 16S rRNA gene sequence of A-SBCA19 *Buchnera* is 98.24% similar to that of *Bu. aphidicola* str. Afa-UT1 (Supplementary Table 3). Put together, the exact biological nature and even the origin of the A-SBCA19 *Buchnera* are not clear and deserve further investigation. Based on 16S rRNA gene sequence, Morrow et al. (2020) reported *Buchnera* in *Diaphorina communis*, as well as in other *Cacopsylla* species and *Cornopsylla rotundiconis*.

### *“Candidatus* Liberibacter asiaticus”

All three San Bernardino county CLas strains from both citrus and ACP were in the PGT-1 group (Dai et al., 2019) (Table 3), suggesting that the San Bernardino strains were still part of the California/Asiatic CLas group. Of particular interest is citrus sample C-SBCA19, which had high plasmid titer (ANC = 20.00) but only partial CLas genome. Considering the lytic nature of Type 1 prophage (Zhang et al., 2011), it is assumed that sample C-SBCA19 could have been collected during the phage induced lytic stage of CLas, i.e., degraded CLas chromosomes and high number of phage particles (containing circular DNA molecules/plasmids).

In summary, a metagenomic pipeline was established for bacteriomic analysis of HLB related ACP and citrus samples from southern California. Ten bacteria including six previously unknown or little known AABacts/CABacts were identified based on draft whole-genome sequences. The whole genome of a CLas strain recently found in San Bernardino County was sequenced. Based on sequence similarity, presence of *Bradyrhizobium* and *Mesorhizobium* could be the source of interference in CLas detection using TaqMan HLBaspr-PCR, particularly at low or zero CLas titer situation.

## Data Availability Statement

The data presented in this study are deposited in the NCBI GenBank repository with Bioproject No. PRJNA704462 and No. PRJNA706130. Genome sequence accession numbers can be found in the article/ Supplementary Material.

## Author Contributions

JC, XD, and JH designed the project, conducted the data analysis, and wrote the manuscript. ZD, ZZ, and PS assisted in data analyses. LK and QX performed the samples collection and DNA preparation. All the authors contributed to the manuscript revision and read and approved the submitted version.

## Conflict of Interest

The authors declare that the research was conducted in the absence of any commercial or financial relationships that could be construed as a potential conflict of interest.

## Footnotes

1 http://sourceforge.net/projects/bbmap

## References

[B1] Aziz R. K. Bartels D. Best A. A. DeJongh M. Disz T. Edwards R. A. (2008). The RAST server: rapid annotations using subsystems technology. *BMC Genomics* 9:75. 10.1186/1471-2164-9-75 18261238 PMC2265698

[B2] Baccari C. Antonova E. Lindow S. (2019). Biological control of Pierce’s disease of grape by an endophytic bacterium. *Phytopathology* 109 248–256. 10.1094/PHYTO-07-18-0245-FI 30540526

[B3] Bao M. Zheng Z. Sun X. Chen J. Deng X. (2020). Enhancing PCR capacity to detect ‘*Candidatus Liberibacter asiaticus*’ utilizing whole genome sequence information. *Plant Dis.* 104 527–532. 10.1094/PDIS-05-19-0931-RE 31790641

[B4] Bausher M. G. Singh N. D. Lee S. B. Jansen R. K. Daniell H. (2006). The complete chloroplast genome sequence of *Citrus sinensis* (L.) Osbeck var ‘Ridge Pineapple’: organization and phylogenetic relationships to other angiosperms. *BMC Plant Biol.* 6:21. 10.1186/1471-2229-6-21 17010212 PMC1599732

[B5] Blacutt A. Ginnan N. Dang T. Bodaghi S. Vidalakis G. Ruegger P. (2020). An in vitro pipeline for screening and selection of citrus-associated microbiota with potential anti - “*Candidatus Liberibacter asiaticus*” properties. *Appl. Environ. Microbiol.* 86:e02883. 10.1128/AEM.02883-19 32086307 PMC7117939

[B6] Blaustein R. A. Lorca G. L. Meyer J. L. Gonzalez C. F. Teplitski M. (2017). Defining the core citrus leaf-and root-associated microbiota: factors associated with community structure and implications for managing huanglongbing (citrus greening) disease. *Appl. Environ. Microbiol.* 83 e210–e217. 10.1128/AEM.00210-17 28341678 PMC5440699

[B7] Bové J. M. (2006). *Huanglongbing*: a destructive, newly-emerging, century-old disease of citrus. *J. Plant Pathol.* 88 7–37. 10.4454/jpp.v88i1.828 32896216

[B8] Camacho C. Coulouris G. Avagyan V. Ma N. Papadopoulos J. Bealer K. (2009). BLAST+: architecture and applications. *BMC Bioinf.* 10:421. 10.1186/1471-2105-10-421 20003500 PMC2803857

[B9] Chen J. Deng X. Sun X. Jones D. Irey M. Civerolo E. (2010). Guangdong and florida populations of ‘*Candidatus Liberibacter asiaticus*’ distinguished by a genomic locus with short tandem repeats. *Phytopathology* 100 567–572. 10.1094/PHYTO-100-6-0567 20465412

[B10] Dai Z. Wu F. Zheng Z. Yokomi R. Kumagai L. Cai W. (2019). Prophage diversity of ‘*Candidatus Liberibacter asiaticus*’ strains in California. *Phytopathology* 109 551–559. 10.1094/PHYTO-06-18-0185-R 30303769

[B11] Deng X. Lopes S. Wang X. Sun X. Jones D. Irey M. (2014). Characterization of “*Candidatus Liberibacter asiaticus*” populations by double-locus analyses. *Curr. Microbiol.* 69 554–560. 10.1007/s00284-014-0621-9 24912994

[B12] Ginnan N. A. Dang T. Bodaghi S. Ruegger P. M. Peacock B. B. McCollum G. (2018). Bacterial and fungal next generation sequencing datasets and metadata from citrus infected with ‘*Candidatus Liberibacter asiaticus*’. *Phytobiomes* 2 64–70. 10.1094/PBIOMES-08-17-0032-A

[B13] Gurevich A. Saveliev V. Vyahhi N. Tesler G. (2013). QUAST: quality assessment tool for genome assemblies. *Bioinformatics* 29 1072–1075. 10.1093/bioinformatics/btt086 23422339 PMC3624806

[B14] Hosmani P. S. Flores-Gonzalez M. Shippy T. Vosburg C. Massimino C. Tank W. (2019). Chromosomal length reference assembly for *Diaphorina citri* using single-molecule sequencing and Hi-C proximity ligation with manually curated genes in developmental, structural and immune pathways. *Biorxiv* [Preprint] 10.1101/869685 bioRxiv: 869685,

[B15] Huang Y. Niu B. Gao Y. Fu L. Li W. (2010). CD-HIT suite: a web server for clustering and comparing biological sequences. *Bioinformatics* 26 680–682. 10.1093/bioinformatics/btq003 20053844 PMC2828112

[B16] Jagoueix S. Bové J. M. Garnier M. (1994). The phloem-limited bacterium of greening disease of citrus is a member of the alpha subdivision of the *Proteobacteria*. *Int. J. Syst. Bacteriol.* 44 379–386. 10.1099/00207713-44-3-379 7520729

[B17] Kolora L. D. Powell C. M. Hunter W. Bextine B. Lauzon C. R. (2015). Internal extracellular bacteria of *Diaphorina citri* Kuwayama (Hemiptera: Psyllidae), the Asian citrus psyllid. *Curr. Microbiol.* 70 710–715. 10.1007/s00284-015-0774-1 25645736

[B18] Konstantinidis K. T. Tiedje J. M. (2005). Genomic insights that advance the species definition for prokaryotes. *Proc. Natl. Acad. Sci*. U.S.A. 102 2567–2572. 15701695 10.1073/pnas.0409727102 PMC549018

[B19] Kumagai L. B. LeVesque C. S. Blomquist C. L. Madishetty K. Guo Y. Woods P. W. (2013). First report of *Candidatus Liberibacter asiaticus* associated with citrus huanglongbing in California. *Plant Dis.* 97 283–283. 10.1094/PDIS-09-12-0845-PDN 30722341

[B20] Langmead B. Salzberg S. L. (2012). Fast gapped-read alignment with bowtie 2. *Nat. Methods* 9:357. 10.1038/nmeth.1923 22388286 PMC3322381

[B21] Li D. Luo R. Liu C. Leung C. Ting H. Sadakane K. (2016). MEGAHIT v1. 0: a fast and scalable metagenome assembler driven by advanced methodologies and community practices. *Methods* 102 3–11. 10.1016/j.ymeth.2016.02.020 27012178

[B22] Li W. Hartung J. S. Levy L. (2006). Quantitative real-time PCR for detection and identification of *Candidatus* Liberibacter species associated with citrus huanglongbing. *J. Microbiol. Methods* 66 104–115. 10.1016/j.mimet.2005.10.018 16414133

[B23] Lodewyckx C. Vangronsveld J. Porteous F. Moore E. R. Taghavi S. Mezgeay M. (2002). Endophytic bacteria and their potential applications. *Crit. Rev. Plant Sci.* 21 583–606. 10.1080/0735-260291044377

[B24] Meng L. Cheng X. Zhang H. (2019). 16S rRNA gene sequencing reveals a shift in the microbiota of *Diaphorina citri* during the psyllid life cycle. *Front. Microbiol.* 10:1948. 10.3389/fmicb.2019.01948 31507561 PMC6716071

[B25] Menzel P. Ng K. L. Krogh A. (2016). Fast and sensitive taxonomic classification for metagenomics with Kaiju. *Nat. Commun.* 7:11257. 10.1038/ncomms11257 27071849 PMC4833860

[B26] Morrow J. L. Om N. Beattie G. A. Chambers G. A. Donovan N. J. Liefting L. W. (2020). Characterization of the bacterial communities of psyllids associated with Rutaceae in Bhutan by high throughput sequencing. *BMC Microbiol*. 20:215. 10.1186/s12866-020-01895-4 32689950 PMC7370496

[B27] Nakabachi A. Ueoka R. Oshima K. Teta R. Mangoni A. Gurgui M. (2013). Defensive bacteriome symbiont with a drastically reduced genome. *Curr. Biol.* 23 1478–1484. 10.1016/j.cub.2013.06.027 23850282

[B28] Pritchard L. Glover R. H. Humphris S. Elphinstone J. G. Toth I. K. (2016). Genomics and taxonomy in diagnostics for food security: soft-rotting enterobacterial plant pathogens. *Anal. Methods* 8 12–24. 10.1039/C5AY02550H

[B29] Sawada H. Kuykendall L. D. Young J. M. (2003). Changing concepts in the systematics of bacterial nitrogen-fixing legume symbionts. *J. Gen. Appl. Microbiol.* 49 155–179. 10.2323/jgam.49.155 12949698

[B30] Shih H. T. Su C. C. Chang C. J. Vargas S. Dai Z. Chen J. (2019). Draft genome sequence of “*Candidatus* Sulcia muelleri” strain KPTW1 from Kolla paulula, a vector of *Xylella fastidiosa* causing Pierce’s disease of grapevine in Taiwan. *Microbiol. Resour. Announc.* 8:e01347. 10.1128/MRA.01347-18 30643878 PMC6328651

[B31] Shin K. van Bruggen A. H. (2018). *Bradyrhizobium* isolated from huanglongbing (HLB) affected citrus trees reacts positively with primers for ‘*Candidatus Liberibacter asiaticus*’. *Eur. J. Plant Pathol.* 151 291–306.

[B32] Song X. B. Peng A. T. Ling J. F. Cui Y. P. Cheng B. P. Zhang L. H. (2019). Composition and change in the microbiome of *Diaphorina citri* infected with *Candidatus Liberibacter asiaticus* in China. *Int. J. Trop. Insect Sci.* 39 283–290. 10.1007/s42690-019-00036-3

[B33] Trivedi P. Duan Y. Wang N. (2010). Huanglongbing, a systemic disease, restructures the bacterial community associated with citrus roots. *Appl. Environ. Microbiol.* 76 3427–3436. 10.1128/AEM.02901-09 20382817 PMC2876436

[B34] Uritskiy G. V. DiRuggiero J. Taylor J. (2018). MetaWRAP—a flexible pipeline for genome-resolved metagenomic data analysis. *Microbiome* 6:158. 10.1186/s40168-018-0541-1 30219103 PMC6138922

[B35] Van Horn C. Dai Z. Sisterson M. Chen J. (2019). Microbiomes of *Xylella fastidiosa* infected grapevine in California. *Am. Phytopathol. Soc. Abstr.* 109:S2.

[B36] Wang X. Tan J. Bai Z. Su H. Deng X. Li Z. (2013). Detection and characterization of miniature inverted-repeat transposable elements in “*Candidatus Liberibacter asiaticus*”. *J. Bacteriol.* 195 3979–3986. 10.1128/JB.00413-13 23813735 PMC3754606

[B37] Wood D. E. Lu J. Langmead B. (2019). Improved metagenomic analysis with Kraken 2. *Genome Biol.* 20:257. 10.1186/s13059-019-1891-0 31779668 PMC6883579

[B38] Wu F. Deng X. Liang G. Huang J. Cen Y. Chen J. (2015a). Whole-genome sequence of “*Candidatus* Profftella armatura” from *Diaphorina citri* in Guangdong, China. *Genome Announc.* 3:e1282. 10.1128/genomeA.01282-15 26543117 PMC4645202

[B39] Wu F. Kumagai L. Cen Y. Chen J. Wallis C. M. Polek M. (2017). Analyses of mitogenome sequences revealed that Asian citrus psyllids (*Diaphorina citri*) from California were related to those from Florida. *Sci. Rep.* 7:10154. 10.1038/s41598-017-10713-3 28860662 PMC5578989

[B40] Wu F. Kumagai L. Liang G. Deng X. Zheng Z. Keremane M. (2015b). Draft genome sequence of “*Candidatus Liberibacter asiaticus*” from a citrus tree in San Gabriel, California. *Genome Announc.* 3:e1508–e1515. 10.1128/genomeA.01508-15 26701083 PMC4691657

[B41] Wu G. A. Prochnik S. Jenkins J. Salse J. Hellsten U. Murat F. (2014). Sequencing of diverse mandarin, pummelo and orange genomes reveals complex history of admixture during citrus domestication. *Nat. Biotechnol.* 32 656–662. 10.1038/nbt.2906 24908277 PMC4113729

[B42] Xu Q. Chen L. Ruan X. Chen D. Zhu A. Chen C. (2013). The draft genome of sweet orange (*Citrus sinensis*). *Nat. Genet.* 45:59. 10.1038/ng.2472 23179022

[B43] Yu F. Bi C. Wang X. Qian X. Ye N. (2018). The complete mitochondrial genome of Citrus sinensis. *Mitochondrial DNA B Resour*. 3 592–593. 10.1080/23802359.2018 33474255 PMC7799571

[B44] Zhang M. Powell C. A. Guo Y. Benyon L. Duan Y. (2013). Characterization of the microbial community structure in ‘*Candidatus Liberibacter asiaticus*’-infected citrus plants treated with antibiotics in the field. *BMC Microbiol.* 13:112. 10.1186/1471-2180-13-112 23701743 PMC3672075

[B45] Zhang S. Flores-Cruz Z. Zhou L. Kang B. H. Fleites L. A. Gooch M. D. (2011). ‘Ca. Liberibacter asiaticus’ carries an excision plasmid prophage and a chromosomally integrated prophage that becomes lytic in plant infections. *Mol. Plant-Microbe Interact.* 24, 458–468. 10.1094/MPMI-11-10-0256 21190436

[B46] Zhang Y. Xu J. Riera N. Jin T. Li J. Wang N. (2017). Huanglongbing impairs the rhizosphere-to-rhizoplane enrichment process of the citrus root-associated microbiome. *Microbiome* 5:97. 10.1186/s40168-017-0304-4 28797279 PMC5553657

[B47] Zheng Z. Bao M. Wu F. Van Horn C. Chen J. Deng X. (2018). A type 3 prophage of ‘*Candidatus Liberibacter asiaticus*’ carrying a restriction-modification system. *Phytopathology* 108 454–461. 10.1094/PHYTO-08-17-0282-R 29192841

[B48] Zheng Z. Deng X. Chen J. (2014b). Whole-genome sequence of “*Candidatus Liberibacter asiaticus*” from Guangdong, China. *Genome Announc.* 2:e00273. 10.1128/genomeA.00273-14 24723715 PMC3983304

[B49] Zheng Z. Deng X. Chen J. (2014a). Draft genome sequence of “*Candidatus Liberibacter asiaticus*” from California. *Genome Announc.* 2:e00999. 10.1128/genomeA.00999-1 PMC4183884 25278540

[B50] Zheng Z. Wu F. Kumagai L. Á Polek M. Deng X. Chen J. (2017). Two ‘*Candidatus Liberibacter asiaticus*’ strains recently found in California harbor different prophages. *Phytopathology* 107 662–668. 10.1094/PHYTO-10-16-0385-R 28398165

[B51] Zheng Z. Xu M. Bao M. Wu F. Chen J. Deng X. (2016). Unusual five copies and dual forms of *nrdB* in “*Candidatus Liberibacter asiaticus*”: Biological implications and PCR detection application. *Sci. Rep.* 6:39020. 10.1038/srep39020 27958354 PMC5154197

