# Supplementary material for: Bacteriomic Analyses of Asian Citrus Psyllid and Citrus Samples Infected With “Candidatus Liberibacter asiaticus” in Southern California and Huanglongbing Management Implications
Source: Front Microbiol. 2021 Jul 2;12:683481. doi: 10.3389/fmicb.2021.683481 (PMC8283493; doi:10.3389/fmicb.2021.683481)
Supplement: Supplementary file 1 [file Data_Sheet_1.docx]

**Supplementary Table 1|** A list of draft genome sequences of ten bacteria [Asian citrus psyllid (ACP)-associated bacteria (AABacts) and citrus-associated bacteria (CABacts)] from seven samples collected in southern California. The sequences were described by five metrics: DGS (draft genome size) in bp (bold), RC% (percentage of DGS/Reference genome size), Total contig in number (before validation / after validation), N50 in bp, and ANC (average nucleotide coverage). The draft genome sequences were acquired using the established pipeline (Table 2) in this study. Data in this table were graphically presented in Fig. 2. Grey area represented no data. The draft genome of AABacts is available in Bioproject No. PRJNA704462 and CABacts is available in Bioproject No. PRJNA706130.

| **Bacterial strain / Reference genome size** |  | **Samples name (Ct values of HLBaspr-PCR)** | | | | | | |
| --- | --- | --- | --- | --- | --- | --- | --- | --- |
|  | Metrics | A-SBCA19 (23.39) | A-SBCA18 (28.62) | A-RSCA17 (29.80) | A-TECA18 (36.81) | A-AHCA17 (23.31) | C-SBCA19 (26.78) | C-AHCA17 (27.52) |
| ‘*Candidatus* Profftella armature’ / 466,568 ^b^ | DGS | **406,686** | **458,046** | **461,651** | **439,465** | **465,454** |  |  |
|  | RC% | 87.17 | 98.17 | 98.95 | 94.19 | 99.76 |  |  |
|  | Total contig | 410/410 | 17/17 | 34/34 | 96/96 | 48/48 |  |  |
|  | N50 | 1,378 | 56,430 | 41,141 | 7,129 | 49,054 |  |  |
|  | ANC | 888.31 | 963.27 | 604.01 | 108.69 | 644.75 |  |  |
|  | GenBank no. | JAGELK000000000 | JAGELM000000000 | JAGELS000000000 | JAGELP000000000 | JAGELV000000000 |  |  |
| ‘*Candidatus* Carsonella ruddii’ / 174,014 ^c^ | DGS | **163,755** | **170,999** | **166,870** | **97,399** | **175,097** |  |  |
|  | RC% | 94.10 | 98.27 | 95.89 | 55.97 | 100.62 |  |  |
|  | Total contig | 169/169 | 9/9 | 15/15 | 132/132 | 5/5 |  |  |
|  | N50 | 1,449 | 29,780 | 18,080 | 993 | 58,565 |  |  |
|  | ANC | 823.44 | 105.93 | 70.66 | 25.29 | 254.47 |  |  |
|  | GenBank no. | JAGELY000000000 | JAGELN000000000 | JAGELT000000000 | JAGELQ000000000 | JAGELW000000000 |  |  |
| *Wolbachia /* 1,216,102 ^d^ | DGS | **1,254,403** | **1,410,235** | **1,422,967** | **1,282,681** | **1,499,370** |  |  |
|  | RC% | 103.15 | 115.96 | 117.01 | 105.47 | 123.29 |  |  |
|  | Total contig | 768/767 | 323/349 | 293/323 | 267/277 | 486/575 |  |  |
|  | N50 | 2,936 | 15,790 | 24,780 | 13,931 | 19,891 |  |  |
|  | ANC | 786.06 | 213.35 | 12037.25 | 2503.84 | 1622.00 |  |  |
|  | GenBank no. | JAGELL000000000 | JAGELO000000000 | JAGELU000000000 | JAGELR000000000 | JAGELX000000000 |  |  |
| ‘*Candidatus* Liberibacter asiaticus’ / 1,227,328 ^a^ | DGS | **1,066,464** | **19,674** | **799,019** |  | **1,222,637** | **98,097** | **182,960** |
|  | RC% | 86.89 | 1.60 | 65.10 |  | 99.62 | 7.99 | 14.90 |
|  | Total contig | 980/980 | 21/21 | 468/468 |  | 86/86 | 162/162 | 361/361 |
|  | N50 | 1524 | 1,463 | 4,076 |  | 43,880 | 653 | 508 |
|  | ANC | 24.35 | 6.33 | 19.78 |  | 74.82 | 11.41 | 3.55 |
|  | GenBank No. (Contig: No) | JAGELZ000000000 (Contig: 1 to 980) | JAGEMA000000000 (Contig:1 to 21) | JAGEMB000000000 (Contig:1 to 468) |  | JAGEMD000000000 (Contig: 1 to 86) | JAFNIF000000000 (Contig: 1 to 162) | JAFNIG000000000 (Contig: 1 to 361) |
| *Buchnera* sp. / 634,931 ^e^ | DGS | **603,974** |  |  |  |  |  |  |
|  | RC% | 95.12 |  |  |  |  |  |  |
|  | Total contig | 261/261 |  |  |  |  |  |  |
|  | N50 | 3,855 |  |  |  |  |  |  |
|  | ANC | 206.43 |  |  |  |  |  |  |
|  | GenBank No. (Contig: No) | JAGELZ000000000 (Contig: 981 to 1241) |  |  |  |  |  |  |
| *Burkholderia* / 7,911,435 ^f^ | DGS | **4,126,133** | **39,424** | **331,041** | **326** | **94,342** | **7,343** |  |
|  | RC% | 52.15 | 0.50 | 4.18 | 0.00 | 1.19 | 0.09 |  |
|  | Total contig | 3187/3232 | 37/43 | 254/278 | 1/1 | 80/92 | 19/21 |  |
|  | N50 | 2699 | 1764 | 2458 | 326 | 1737 | 344 |  |
|  | ANC | 52.60 | 5.18 | 8.52 | 5.69 | 3.76 | 5.81 |  |
|  | GenBank No. (Contig: No) | JAGELZ000000000 (Contig: 1242 to 4428) | JAGEMA000000000 (Contig: 22 to 58) | JAGEMB000000000 (Contig: 469 to 722) | JAGEMC000000000 (Contig: 1) | JAGEMD000000000 (Contig: 87 to 166) | JAFNIF000000000 (Contig: 163 to 181) |  |
| *Paraburkholderia*  / 8,093,536 ^g^ | DGS | **336,610** | **145,937** | **127,026** |  | **73,479** |  |  |
|  | RC% | 3.83 | 1.67 | 1.47 |  | 0.84 |  |  |
|  | Total contig | 155/155 | 99/100 | 99/99 |  | 42/42 |  |  |
|  | N50 | 4548 | 7368 | 3068 |  | 3238 |  |  |
|  | ANC | 81.54 | 5.62 | 6.43 |  | 8.11 |  |  |
|  | GenBank No. (Contig: No) | JAGELZ000000000 (Contig: 4429 to 4583) | JAGEMA000000000 (Contig: 59 to 157) | JAGEMB000000000 (Contig: 723 to 821) |  | JAGEMD000000000 (Contig: 167 to 208) |  |  |
| *Pseudomonas* /  4,547,930 ^h^ | DGS | **3,684,509** | **31,773,933** | **850,004** | **65,326** | **506,751** |  | **840** |
|  | RC% | 64.60 | 557.07 | 14.90 | 1.14 | 8.88 |  | 0.01 |
|  | Total contig | 2,543 / 2,568 | 8,639 / 11,062 | 473 / 477 | 95 / 95 | 560 / 568 |  | 2 / 2 |
|  | N50 | 3,219 | 10,284 | 4,330 | 752 | 1,150 |  | 490 |
|  | ANC | 45.60 | 115.65 | 5.25 | 9.21 | 6.27 |  | 3.4 |
|  | GenBank No. (Contig: No) | JAGELZ000000000 (Contig: 4584 to 7126) | JAGEMA000000000 (Contig: 158 to 8796) | JAGEMB000000000 (Contig: 822 to 1294) | JAGEMC000000000 (Contig: 2 to 96) | JAGEMD000000000 (Contig: 209 to 768) |  | JAFNIG000000000 (Contig: 362 to 363) |
| *Bradyrhizobium* / 8,003,090 ^i^ | DGS | **14,028** | **464,062** | **26,967** | **46,684** | **255,059** | **7,698** |  |
|  | RC% | 0.18 | 5.80 | 0.34 | 0.58 | 3.19 | 0.10 |  |
|  | Total contig | 16 / 16 | 447 / 460 | 29 / 29 | 39 / 41 | 342 / 367 | 27 / 27 |  |
|  | N50 | 1,195 | 1,663 | 1,440 | 2,049 | 826 | 270 |  |
|  | ANC | 8.10 | 4.87 | 7.48 | 45.79 | 8.48 | 2.88 |  |
|  | GenBank No. (Contig: No) | JAGELZ000000000 (Contig: 7127 to 7142) | JAGEMA000000000 (Contig: 8797 to 9243) | JAGEMB000000000 (Contig: 1295 to 1323) | JAGEMC000000000 (Contig: 97 to 135) | JAGEMD000000000 (Contig: 769 to 1110) | JAFNIF000000000 (Contig: 182 to 208) |  |
| *Mesorhizobium* / 5,731,152 ^j^ | DGS | **1,080** | **5,990,225** | **21,714** | **127,837** | **219,625** |  |  |
|  | RC% | 0.02 | 104.52 | 0.38 | 2.23 | 3.83 |  |  |
|  | Total contig | 2 / 2 | 2,277 / 2,360 | 24 / 25 | 116 / 116 | 294 / 300 |  |  |
|  | N50 | 673 | 10,293 | 1,061 | 1,748 | 810 |  |  |
|  | ANC | 13.00 | 19.73 | 4.06 | 3.76 | 5.81 |  |  |
|  | GenBank No. (Contig: No) | JAGELZ000000000 (Contig: 7143 to 7144) | JAGEMA000000000 (Contig: 9244 to 11520) | JAGEMB000000000 (Contig: 1324 to 1347) | JAGEMC000000000 (Contig: 136 to 251) | JAGEMD000000000 (Contig: 1111 to 1404) |  |  |

*a NC_012985, ‘Candidatus Liberibacter asiaticus’ psy62*

*b NZ_CP041281.1, ‘Candidatus Profftella armature’ JRPAMB3*

*c NZ_CP041245.1, ‘Candidatus Carsonella ruddii’ JRPAMB3*

*d NZ_AMZJ01000000 / Wolbachia sp. wDi*

*e NZ_CP042427 / Buchnera aphidicola Afa-UT1*

*f CADEUK010000000, Burkholderia cenocepacia J2315*

*g NC_010681, Paraburkholderia phytofirmans PsJn;*

*h NC_015740, Pseudomonas Stutzeri CGMCC 1.1803*

*i NZ_CP025113, Bradyrhizobium sp. SK17*

*j NZ_CP044218, Mesorhizobium terrae NIBRBAC 0005000504*

**Supplementary Table 2|** A list of draft genome sequences of Asian citrus psyllid (ACP) mitochondria, citrus mitochondria, and citrus chloroplast from seven samples collected from southern California. The sequences were described by five metrics: DGS (draft genome size) in bp (bold), RC% (percentage of DGS/Reference genome size), Total contig in number (before validation / after validation), N50 in bp, and ANC (average nucleotide coverage). The draft genome sequences were acquired using the established pipeline (Table 2) in this study. Data in this table were graphically presented in Fig. 2. Grey area represented no data.

| **Organelle / Reference genome size / Accession number** |  | **Sample** | | | | | | |
| --- | --- | --- | --- | --- | --- | --- | --- | --- |
|  | **Selected metrics** | A-SBCA19 | A-SBCA18 | A-RSCA17 | A-TECA18 | A-AHCA17 | C-SBCA19 | C-AHCA17 |
| ACP Mitochondria /15,027 bp / KY426014 | DGS | **14,340** | **15,114** | **15,116** | **14,964** | **15,455** |  |  |
|  | RC% | 95.43 | 100.58 | 100.59 | 99.58 | 102.85 |  |  |
|  | Total contig | 14 | 1 | 1 | 1 | 1 |  |  |
|  | N50 | 1,629 | 15,114 | 15,116 | 14,964 | 15,455 |  |  |
|  | ANC | 3672.00 | 4765.40 | 8149.11 | 3649.79 | 63,586.00 |  |  |
| Citrus mitochondria / 640,906 bp / NC_037463 | DGS |  |  |  |  |  | **615,392** | **614,800** |
|  | RC% |  |  |  |  |  | 96.02 | 95.93 |
|  | Total contig |  |  |  |  |  | 135 | 33 |
|  | N50 |  |  |  |  |  | 7,355 | 88,694 |
|  | ANC |  |  |  |  |  | 3,910.14 | 6,619.17 |
| Citrus chloroplast / 160,129 / DQ864733 | DGS |  |  |  |  |  | **152,061** | **133,385** |
|  | RC% |  |  |  |  |  | 94.96 | 83.30 |
|  | Total contig |  |  |  |  |  | 39 | 12 |
|  | N50 |  |  |  |  |  | 7,366 | 34,157 |
|  | ANC |  |  |  |  |  | 5,502.01 | 10,039.04 |

**Supplementary Table 3 |** BLAST comparisons between draft genome sequence (DGS, 603,974 bp) of *Buchnera* sp. A-SBCA19 and complete genome sequence of *Buchnera aphidicola* Afa-UT1 (NZ_CP042427, 634,931 bp). The two bacteria had an ANI of 86.94.

| **Locus** | **Description** | **Gene size (bp)** | | **Similarity ^a^** |
| --- | --- | --- | --- | --- |
|  |  | A-SBCA19 | Afa-UT1 |  |
| *rrs* | 16S rRNA | 1,537 | 1,548 | 98.24% |
| *aspS* | Aspartate—tRNA ligase | 1,760 | 1,761 | 84.89% |
| *gyrA* | DNA gyrase subunit A | 2,514 | 2,520 | 88.80% |
| *ompA* | Outer membrane porin A | 1,026 | 1,029 | 88.92% |

*^a^ BLASTn parameters: Word size = 11; e-value > 1E-64.*
